# Sex‐Specific Associations Between Alcohol Consumption and Incidence of Hypertension: A Systematic Review and Meta‐Analysis of Cohort Studies

**DOI:** 10.1161/JAHA.117.008202

**Published:** 2018-06-27

**Authors:** Michael Roerecke, Sheldon W. Tobe, Janusz Kaczorowski, Simon L. Bacon, Afshin Vafaei, Omer S. M. Hasan, Rohin J. Krishnan, Amidu O. Raifu, Jürgen Rehm

**Affiliations:** ^1^ Institute for Mental Health Policy Research Centre for Addiction and Mental Health (CAMH) Toronto Ontario Canada; ^2^ Dalla Lana School of Public Health (DLSPH) University of Toronto Ontario Canada; ^3^ Institute of Medical Science University of Toronto Ontario Canada; ^4^ Department of Psychiatry University of Toronto Ontario Canada; ^5^ Department of Medicine University of Toronto Ontario Canada; ^6^ PAHO/WHO Collaborating Centre for Addiction and Mental Health Toronto Ontario Canada; ^7^ Department of Family and Emergency Medicine Université de Montréal Quebec Canada; ^8^ CRCHUM (University of Montreal Hospital Research Centre) Montreal Quebec Canada; ^9^ Institute for Clinical Psychology and Psychotherapy Technische Universität Dresden Dresden Germany; ^10^ Campbell Family Mental Health Research Institute CAMH Toronto Ontario Canada; ^11^ Northern Ontario School of Medicine Sudbury Ontario Canada; ^12^ Department of Exercise Science Concordia University Montreal Quebec Canada; ^13^ Montreal Behavioural Medicine Centre CIUSSS‐NIM Montreal Quebec Canada

**Keywords:** alcohol, cohort studies, hypertension, meta‐analysis, systematic review, Epidemiology, Risk Factors, Hypertension

## Abstract

**Background:**

Although it is well established that heavy alcohol consumption increases the risk of hypertension, the risk associated with low levels of alcohol intake in men and women is unclear.

**Methods and Results:**

We searched Medline and Embase for original cohort studies on the association between average alcohol consumption and incidence of hypertension in people without hypertension. Random‐effects meta‐analyses and metaregressions were conducted. Data from 20 articles with 361 254 participants (125 907 men and 235 347 women) and 90 160 incident cases of hypertension (32 426 men and 57 734 women) were included. In people drinking 1 to 2 drinks/day (12 g of pure ethanol per drink), incidence of hypertension differed between men and women (relative risk_women vs men_=0.79; 95% confidence interval, 0.67–0.93). In men, the risk for hypertension in comparison with abstainers was relative risk=1.19 (1.07–1.31; I^2^=59%), 1.51 (1.30–1.76), and 1.74 (1.35–2.24) for consumption of 1 to 2, 3 to 4, and 5 or more standard drinks per day, respectively. In women, there was no increased risk for 1 to 2 drinks/day (relative risk=0.94; 0.88–1.01; I^2^=73%), and an increased risk for consumption beyond this level (relative risk=1.42; 1.22–1.66).

**Conclusions:**

Any alcohol consumption was associated with an increase in the risk for hypertension in men. In women, there was no risk increase for consumption of 1 to 2 drinks/day and an increased risk for higher consumption levels. We did not find evidence for a protective effect of alcohol consumption in women, contrary to earlier meta‐analyses.

**Clinical Perspective**

**What Is New?**

- This is the first meta‐analysis based on high‐quality cohort studies on the relationship between different levels of alcohol consumption and risk for incident hypertension.
- We investigated the risk for hypertension separately for men and women in people who did not have hypertension at baseline.
- The risk for hypertension in former drinkers was similar to that of lifetime abstainers.
- We found that, compared with nondrinkers, the risk for hypertension was increased at all levels of alcohol consumption in men. Contrary to earlier meta‐analyses, we did not find a protective effect of low levels of alcohol consumption in women.

**What Are the Clinical Implications?**

- The findings support sex‐specific drinking guidelines with regard to risk for hypertension. These guidelines may be revised to indicate the increased risk for any alcohol consumption in men.
- Alcohol consumption should be assessed at the primary care level whenever there is elevated blood pressure.
- Changing clinical practice promises to reduce substantial mortality and burden of disease associated with both alcohol consumption and hypertension.

## Introduction

Hypertension (raised blood pressure [BP], >140 mm Hg systolic BP, and/or >90 mm Hg diastolic BP) ranks as the third‐most important risk factor for global burden of disease,^1^ responsible for considerable and increasing noncommunicable diseases burden and mortality.^1^, ^2^ This condition affects more than 1 billion people worldwide with a global prevalence of close to 20%. Despite decreases in raised BP mainly in higher income countries, in part attributed to improved detection and treatment,^3^ global prevalence of hypertension has been increasing and is predicted to further increase in the next decade.^1^, ^2^ In 2015, hypertension was responsible for 10.7 (95% confidence interval [CI], 9.6–11.8) million deaths and 211.8 (95% CI, 192.7–231.1) million disability‐adjusted life years globally.^1^

Hypertension is largely a by‐product of modern lifestyle factors such as lack of physical activity,^4^ unhealthy diet (in particular, salt intake^4^), or consumption of alcohol.^5^ In fact, some guidelines for clinical management, including those from the National Institute for Health and Care Excellence, stipulate that all patients undergoing assessment or treatment for hypertension should receive initial and periodic lifestyle advice, which includes ascertaining their level of alcohol consumption and encouraging a reduced intake if they drink hazardously or heavily.^6^, ^7^ The American Heart Association guidelines for the prevention and treatment of high BP recommend limiting daily alcohol intake to 2 or less drinks for men and 1 or less drinks for women.^8^

The relationship between alcohol consumption and hypertension was first reported by Lian^9^ researching French soldiers serving in World War II. He found a dose‐response association with a 4‐fold increase between drinkers with the lowest (up to 2 L of wine per day) and highest (>3 L of wine per day plus aperitifs) levels of consumption. Numerous studies since then have confirmed the association between heavy drinking and development of hypertension.^10^ However, the association between light‐to‐moderate drinking and hypertension is still disputed,^11^, ^12^ despite a number of meta‐analyses^13^, ^14^ and countless reviews (overview of recent reviews^15^). The association may also depend on sex, which could be related to differential alcohol metabolism^16^ or drinking patterns.^17^

In part because there have been a number of studies since the last systematic review including a meta‐analysis,^14^ and in part because the techniques for conducting meta‐analyses have expanded considerably in recent years,^18^ we conducted a systematic review and meta‐analysis with the explicit aim to restricting our review to studies above a high‐quality threshold and to explore potential influencing factors by stratification by sex and metaregression. This review intends not only to produce improved sex‐specific estimates for comparative risk assessments within the Global Burden of Disease studies^19^ and for the Global Status Reports of the World Health Organization,^20^ but also provide much needed evidence for hypertension‐specific drinking guidelines.

## Methods

All data are from publicly available sources.

### Search Strategy and Selection Criteria

Following the meta‐analysis of observational studies in epidemiology checklist,^21^ we conducted a systematic electronic literature search using Medline and Embase from inception to April 3, 2017 for keywords and MeSH terms relating to alcohol consumption, hypertension, and observational studies (Table S1). Additionally, we searched reference lists of identified articles and published meta‐analyses and reviews. Inclusion criteria were as follows:

1. Full‐text article with original cohort data (including nested case‐control studies) examining the association between total alcohol consumption and incidence of hypertension.
2. Participants with hypertension at baseline were excluded.
3. Analyses were adjusted or matched for age at baseline.
4. Incidence for at least 2 quantitatively defined categories of average alcohol consumption in addition to nondrinkers, or incidence for former drinkers in relation to lifetime abstainers were reported.
5. Results were sex specific.

For a continuous nonlinear dose‐response meta‐analysis, results for at least 3 drinking groups in addition to nondrinkers had to be reported. We did not apply language restrictions. Authors were contacted for clarification and missing data. Two reviewers independently excluded articles based on title and abstract or full text, and abstracted the data. Any discrepancies were resolved in consultation with a third reviewer.

### Data Extraction

From all relevant articles, we extracted authors’ names, year of publication, country, calendar year(s) of baseline examination, follow‐up period, setting of the study, assessment of hypertension status, age (mean or median) at baseline, sex, number of observed incident hypertension cases among participants by drinking group, number of total participants by drinking group, specific adjustment or stratification for potential confounders, and adjusted measures of effect (relative risks [RRs], odds ratios, and hazard ratios) and their confidence intervals or standard errors. Risk estimates by sex and race/ethnicity were treated as independent samples. As a result, multiple articles and estimates from the same study^22^, ^23^, ^24^ were included, but each case of incident hypertension was used only once in each of the analyses conducted. If necessary, effect sizes within studies were recalculated to contrast alcohol consumption categories against nondrinkers.^25^ Because incidence of hypertension was not rare, we transformed odds ratios to RRs based on the formula described by Zhang and Yu.^26^ Hazard ratios and RRs were treated as equivalent measures of risk.

### Exposure and Outcome Assessment

Consolidating exposure measures across primary studies involved a 2‐step process. First, among drinkers, we converted reported alcohol intake categories in primary studies into an average of pure alcohol in g/d using the midpoints (mean or median) of reported drinking group categories. For open‐ended categories, we added three quarters of the second‐highest category's range to the lower limit of the open‐ended category of alcohol intake if the mean was not reported. Standard drinks vary by country, with 1 standard drink containing ≈8 to 14 g of pure alcohol.^27^ We used reported conversion factors when standard drinks were the unit of measurement to convert all measures to g/d. Then, for reporting of our analyses, we considered categories with a mean of up to 12 g of pure ethanol as 1 standard drink for a global representation. Qualitative descriptions, such as “social” or “frequent” drinkers with no clear total alcohol intake in g/d, were excluded.

Because of the changing definitions of hypertension over time, we defined hypertension status at baseline and incident cases of hypertension as defined in the primary studies (typically assessed as taking antihypertensive medications or as mean systolic BP at baseline >140 mm Hg).

### Quality Assessment

Most quality scores are tailored for meta‐analyses of randomized trials of interventions,^28^, ^29^, ^30^ and many criteria do not apply to epidemiological studies examined in this study. Additionally, quality score use in meta‐analyses remains controversial.^31^, ^32^, ^33^ As a result, study quality was incorporated by including quality components, such as study design, measurement of alcohol consumption and hypertension, adjustment for age, and sex‐specific RRs, in the inclusion and exclusion criteria and further by investigating potential heterogeneity in metaregression models and several subgroup analyses. We used the most adjusted RR reported and the most comprehensive data available for each analysis, and gave priority to estimates where lifetime abstainers were used as the risk reference group.

In a formal risk of bias analysis, we used the Cochrane risk of bias tool for nonrandomized studies (ROBINS‐I)^34^ to assess risk of bias in primary studies. We rated the evidence for the association between alcohol consumption and incidence of hypertension based on the Grades of Recommendation, Assessment, Development and Evaluation system.^35^

### Statistical Analyses

In analyses using standard drinks as the exposure measure, RRs were pooled with inverse‐variance weighting using DerSimonian‐Laird random‐effect models to allow for between‐study heterogeneity.^36^ Small‐study bias was examined using Egger's regression‐based test.^37^ Variation in the effect size because of heterogeneity between‐studies was quantified using the I^2^ statistic.^38^ Between‐study heterogeneity was investigated with random‐effects metaregressions.^39^

Using studies that reported data for 4 or more alcohol intake groups, we conducted 2‐stage restricted cubic spline regression in multivariate metaregression models taking into account the variance‐covariance matrix for risk estimates derived from 1 reference group^40^, ^41^ to calculate continuous nonlinear dose‐response curves for total alcohol consumption (g/d) in relation to abstainers. All meta‐analytical analyses were conducted on the natural log scale of the RRs (and hazard ratios) in Stata statistical software version 14.2 (Stata LP, College Station, TX).

## Results

### Literature Search and Study Characteristics

**Figure 1:**
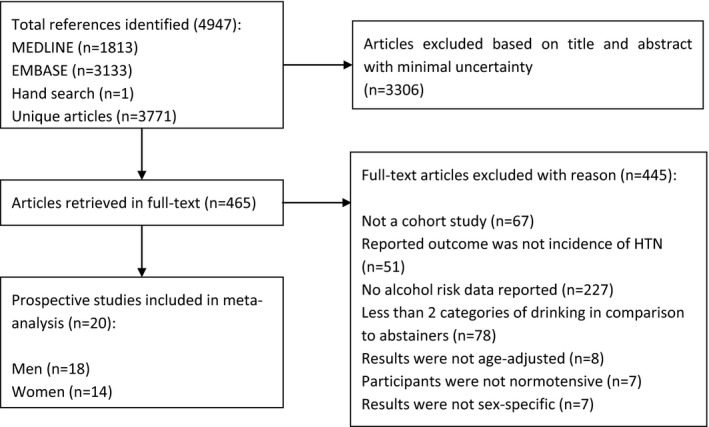
Flowchart of study selection.

Of 3771 identified references, 465 were reviewed in full text. In total, data from 20 reports from 18 studies were used in the analysis (Figure 1). Nine reports were from the United States,^22^, ^23^, ^24^, ^42^, ^43^, ^44^, ^45^, ^46^, ^47^ 4 from Japan,^48^, ^49^, ^50^, ^51^ 2 from China,^52^, ^53^ and 1 each from Germany,^54^ South Korea,^55^ Finland,^56^ Turkey,^57^ and Thailand^58^ (Table). Several reports from the Nurses’ Health Study^42^, ^44^, ^45^, ^46^ were included, but any 1 case of incident hypertension was included only once in any particular analysis. Overall, data from 361 254 participants (125 907 men and 235 347 women) and 90 160 incident cases of hypertension (32 426 men and 57 734 women) were analyzed. Mean age at baseline among men ranged from 25 to 57 years with a weighted mean of 47.1 years (median=50 years), with mean follow‐up duration of 5.3 years (median=4; range, 3.9–20.0). In women, mean age ranged from 25 to 60 years with a weighted mean of 46.7 years (median=54), with mean follow‐up duration of 7.3 years (median=4; range, 3.9–20.0). Most studies were well adjusted for potential confounders; 1 study was adjusted only for age.^55^

**Table 1:** Characteristics of 20 Cohort Studies Investigating Sex‐Specific Incidence of Hypertension by Alcohol Intake in People Without Hypertension at Baseline, 1989–2017

Reference	Baseline Year(s), Setting	Baseline Hypertension Status, Sex, Age (y), Country	Design, Cases (No.), Participants (No.), Follow‐up Time (y)	Alcohol Assessment at Baseline	Assessment of Hypertension During Follow‐up	Adjustments
Ascherio et al, 1996^42^	1984. White female nurses from the NHS I (Nurses’ Health Study). Baseline exclusions: pregnant for at least 6 months, use of antihypertensive drugs, on a special diet, high BP (140/90 mm Hg), MI, coronary artery surgery, stroke, angina pectoris, diabetes mellitus, and all cancers except nonmelanoma skin cancer	Normotensive, W, 38 to 63, USA	Cohort, 2526, 41 541, 4	Lifetime abstainers, current drinkers: (0.1–9, 10–19, 20–29, ≥30) g/d	Self‐reported physician diagnosed hypertension (140/90 mm Hg), confirmed by review of medical record in a subsample n=100	Age, BMI
Bae and Ahn, 2002^55^	1992. Healthy Korean men from the Seoul Cohort Study, and beneficiaries of the Korea Medical Insurance Corporation (KMIC)	Normotensive, M, 40 to 59, South Korea	Nested case control, 236, 1116, 4	Current abstainers, current drinkers: (1–70, 71–280, 281–560, >560) g/wk	Review of medical records through the hospital survey, use of antihypertensive drugs, self‐reporting on telephone, and clinical assessment of hypertension (140/90 mm Hg). JNC VI criteria for hypertension were used.	Frequency matched on age
Bai et al, 2017^53^	2000. CHNS (China Health and Nutrition Survey). A multistage random cluster sampling in Heilongjiang, Liaoning, Jiangsu, Shandong, Henan, Hubei, Hunan, Guizhou, and Guangxi. Baseline exclusions: <18 or >60 years age, missing data on BP, hypertension at baseline, taking antihypertensive medication, existing diagnosis of diabetes mellitus, MI, stroke	Normotensive, M,W, 18 to 60, China	Cohort, 1147, 2751, 11	Lifetime abstainers, former drinkers, current drinkers (0.1–10.0, 10.1–25.0, >25.0) g/d	Having an average SBP⩾140 mm Hg, an average DBP ≥90 mm Hg, currently undergoing treatment with an antihypertensive medication, or having received a previous diagnosis by a physician	Age, income, employment status, education, province, urban or rural, DASH score, physical activity, BMI, smoking
Banda et al, 2010^43^	1974–2003. Predominantly white males from the ACLA (Aerobics Center Longitudinal Study), well‐educated, middle and upper socioeconomic class, free of CVD, cancer, and hypertension at baseline	Normotensive, M, 44 (20–82), USA	Cohort, 1959, 14 568, 10.7	Current abstainers, current drinkers: (1–14, >14) drinks/week	Self‐reported physician diagnosed hypertension (140/90 mm Hg) through health survey	Age (single year), examination year, survey response pattern, resting SBP and DBP, diabetes mellitus, and family history of hypertension, BMI, smoking, physical activity, and cardiorespiratory fitness
Diederichs and Neuhauser, 2017^54^	1998. Adult population from the GNHIES (German National Health Interview and Examination Survey), free of hypertension at baseline	Normotensive, M,W, 18 to 79, Germany	Cohort, 585, 2231, 11.9	Men: Current abstainers, current drinkers: (<20, ≥20) g/d Women: Current abstainers, current drinkers: (<10, ≥10) g/d	Clinical assessment of hypertension (140/90 mm Hg), by taking average of the last 2 of 3 BP readings, each 3 minutes apart, after an initial rest of 5 minutes	Age, socioeconomic status, SBP, DBP, BMI, diabetes mellitus, hyperlipidemia, smoking, physical activity, community size, regions, health insurance
Forman et al, 2009^44^	1991. Female nurses from the NHS II (Nurses’ Health Study), with normal BP (≤120/80 mm Hg) and free of diabetes mellitus, CVD, or cancer at baseline	Normotensive, W, 36, USA	Cohort, 10 152, 83 882, 14	Current abstainers, current drinkers: (0.1–5, 5.1–10, 10.1–15, 15.1–29.9, ≥30) g/d	Self‐reported hypertension (140/90 mm Hg) confirmed by medical record review in a subsample n=147	Age, race, family history of hypertension, use of oral contraceptive pills, smoking status, quintile of DASH score, vigorous exercise, BMI, supplemental folic acid intake, frequency of acetaminophen use, frequency of NSAID use, frequency of aspirin use
Fuchs et al, 2001^23^	1988. Black and white adults from the ARIC (Atherosclerosis Risk in Communities) Study, free of hypertension and CHD at baseline and alive throughout the follow‐up	Normotensive, M,W, 45 to 64, USA	Cohort, 1243, 8334, 6	Current abstainers, current drinkers: (1–209, ≥210) g/wk	Clinical assessment of hypertension (140/90 mm Hg) by taking the average of the second and third reading after 5 minutes of rest	Age, BMI, education, physical activity, and diabetes mellitus. Stratified by race
Halanych et al, 2010^22^	1985. Young black and white men and women from the CARDIA (Coronary Artery Risk Development in Young Adults) Study, free of hypertension at baseline	Normotensive, M,W, 24.8, USA	Cohort, 1022, 4711, 20	Men: Never drinkers, former drinkers, current drinkers: (0–7, 7–14, >14) drinks/week. Women: Never drinkers, former drinkers, current drinkers: (0–4, 4–7, >7) drinks/week	Clinical assessment of hypertension (140/90 mm Hg) as the mean of the second and third BP measurements, or use of antihypertensive drugs	Age, family history of hypertension, BMI (continuous), smoking status, race, sex, education, income, difficulty paying for basics, and difficulty paying for medical care. Stratified by race
Nakanishi et al, 2001^48^	1990. Japanese male office workers from the Takenaka Corporation in Osaka, free of hypertension at baseline	Normotensive, M, 45.7, Japan	Cohort, 458, 1130, 9	Current abstainers, current drinkers: (0.1–22.9, 23–45.9, 46–68.9, ≥69) g/d	Clinical assessment of hypertension (140/90 mm Hg) after a 5‐minute rest, and/or receipt of antihypertensive medications	Age, BMI, cigarette smoking, total cholesterol level, triglyceride level, and fasting plasma glucose level at study entry
Nakanishi et al, 2002^49^	1996. Japanese male office workers, free of hypertension at baseline	Normotensive, M, 23 to 59, Japan	Cohort, 964, 3784, 4	Current abstainers, current drinkers: (<12, 12–22, 23–45, ≥46) g/d	Clinical assessment of hypertension (140/90 mm Hg) after a 5‐minute rest, in a seated position, or self‐report of antihypertensive medication use on an annual survey	Age, BMI, family history of hypertension, cigarette smoking, total cholesterol level, triglyceride level, fasting plasma glucose level
Niskanen et al, 2004^56^	1987–1989. General population from the Kuopio Ischemic Heart Disease Risk Factor Study, free of hypertension and diabetes mellitus at baseline	Normotensive, M, 51, Finland	Cohort, 124, 379, 11	Current abstainers, current drinkers: (1–83, ≥84) g/wk	Clinical assessment of hypertension (140/90 mm Hg) by taking the average of 2 BP readings while sitting with a 5‐minute interval of rest in between	Age, smoking, socioeconomic status, leisure‐time physical activity, CVD, dietary factors (saturated fat, sodium, potassium, fruits, vegetables), baseline SBP, waist girth, concentrations of insulin, glucose, HDL cholesterol, changes in waist girth, smoking, alcohol intake during follow‐up
Ohmori et al, 2002^50^	1978. Subrural Japanese men from the Hisayama Study, with normal BP and free from CVD at baseline	Normotensive, M, 53, Japan	Cohort, 101, 433, 10	Never drinkers, former drinkers, current drinkers: (<23, 23–45, ≥46) g/d	Clinical assessment of hypertension (140/90 mm Hg) on at least 2 occasions in different examinations	Age, BMI
Okubo et al, 2014^51^	1993–2004. General Japanese population from the IPHS (Ibarakai Prefectural Health Study) underwent community‐based health checkups, free of hypertension, history of heart disease or stroke at baseline. Those who had stopped drinking alcohol were also excluded.	Normotensive, M,W, 56.9, Japan	Cohort, 45 428, 115 736, 3.9 (1–18)	Current abstainers, current drinkers: (1.0–19.9, 20.0–39.9, 40.0–59.9, ≥60) g/d	Clinical assessment of hypertension (140/90 mm Hg) by taking a BP measurement after 5 minutes of rest by a trained nurse	Age, BMI, SBP, cholesterol, HDL‐cholesterol level, triglyceride level (log), antidyslipidemic medication use, blood glucose level, anti–diabetes mellitus medication use, smoking status. Stratified by age
Onat et al, 2008^57^	1997. General population from the Turkish Adult Risk Factor Study, free of hypertension at baseline	Normotensive, M,W, 47.6, Turkey	Cohort, 645, 2683, 9	Current abstainers, current drinkers: (1–3, >3) drinks/day	Clinical assessment of hypertension (140/90 mm Hg), while sitting, average of 2 readings, at least 3 minutes apart	Age, physical activity, smoking status, lipid‐lowering therapy, hormone replacement therapy (only in women)
Peng et al, 2013^52^	2006. Current and retired coal mine workers from the Kailuan study, free of hypertension, stroke, transient ischemia attack, MI, and cancer (except nonmelanoma skin cancer) at baseline	Normotensive, M, 49.9, China	Cohort, 9151, 32 389, 4	Current abstainers, current drinkers: (1–24, 25–49, 50–99, 100–149, ≥150) g/d	Cases had to meet 2 of the 3 criteria: self‐report of newly diagnosed hypertension; self‐report of newly initiated antihypertensive treatment; on‐site measured SBP at least 140 mm Hg and DBP at least 90 mm Hg, or either of them, then confirmed by at least 2 follow‐up BP measurements	Age, exercise, smoking status, type of work (mental or physical), salt intake, BMI, history of high cholesterol, history of diabetes mellitus
Sesso et al, 2008^47^	1992. Male physicians (age, 40–84) from the PHS (Physicians’ Health Study) and female health professionals (age, ≥45) from the WHS (Women's Health Study), who were postmenopausal or not intending to become pregnant. All participants were also free of hypertension, stroke, MI, transient ischemic attack, and cancer (except nonmelanoma skin cancer) at baseline	Normotensive, M,W, 40 to 84 (PHS), ≥45 (WHS), USA	Cohort, 14 692, 42 303, 10.9 (WHS) and 21.8 (PHS)	Men: Rarely or never drinkers, current drinkers: (1–3) drinks/mo (1, 2–4, 5–6) drinks/wk (1, ≥2) drinks/day Women: Rarely or never drinkers, current drinkers: (1–3) drinks/mo (1, 2–4, 5–6) drinks/wk (1, 2–3, ≥4) drinks/d	Self‐reported hypertension (140/90 mm Hg), not necessarily physician diagnosed, and use of antihypertensive drugs	Age, exercise, parental history of MI, aspirin use, carotene, vitamin E treatment, postmenopausal status, smoking status, hormone replacement therapy, BMI, history of high cholesterol, history of diabetes mellitus
Thawornchaisit et al, 2013^58^	2005. University students from the TCS (Thai Cohort Study), free of hypertension at baseline	Normotensive, M,W, 31, Thailand	Cohort, 578, not reported, 4	Never drinkers, former drinkers	Self‐reported physician diagnosed hypertension	Age, marital status, education, income, BMI category, underlying diseases, personal behaviors
Wang et al, 2011^24^	1994–1998. Postmenopausal black and white women from the Women's Health Initiative Observational Study	Normotensive, W, 60.8, USA	Nested case control, 800, 1600, 5.9	Never drinkers, former drinkers, current drinkers: (<1, 1–7, ≥7) drinks/week	Clinical assessment of hypertension (140/90 mm Hg), after 5 minutes of rest, and mean of 2 readings 30 seconds apart, or self‐report of use of antihypertensive drugs on an annual questionnaire	Individually matched on age, ethnicity, clinical center, and time of enrollment
Witteman et al, 1989,^45^ 1990^46^	1980. Female nurses from the NHS I, free of antihypertensive medication, pregnancy in the last 6 months, high BP, MI, angina pectoris, diabetes mellitus, all cancers except nonmelanoma skin cancer, and any special diet at baseline	Normotensive, W, 34 to 59, USA	Cohort, 3275, 58 218, 4	Current abstainer, current drinkers: (0.1–9, 10–19, 20–29, ≥30) g/d. Stratified by age^46^	Self‐reported physician diagnosed hypertension (140/90 mm Hg)	Age, Quetelet's index, and intakes of calcium, magnesium, potassium, and fiber. Age‐stratified data^46^ were adjusted for Quetlet's index.

BMI indicates body mass index; BP, blood pressure; CHD, congestive heart disease; CVD, cardiovascular disease; DASH, Dietary Approaches to Stop Hypertension; DBP, diastolic blood pressure; HDL, high‐density lipoprotein; JNC VI, sixth report of the Joint National Committee on Prevention, Detection, Evaluation, and Treatment of High Blood Pressure; M, men; M,W, men and women stratified; MI, myocardial infarction; NSAID, nonsteroidal anti‐inflammatory drug; SBP, systolic blood pressure; W, women.

### Meta‐Analyses

**Figure 2:**
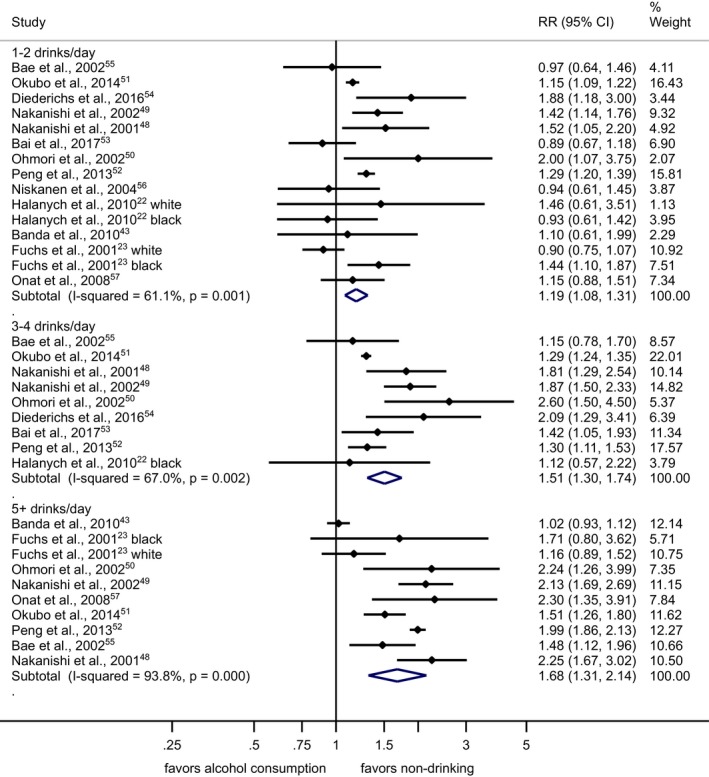
Incidence of hypertension in men by alcohol intake at baseline in standard drinks compared with abstainers in cohort studies, 1989–2017. 1 standard drink=12 g of pure ethanol per day. CI indicates confidence interval; RR, relative risk.

**Figure 3:**
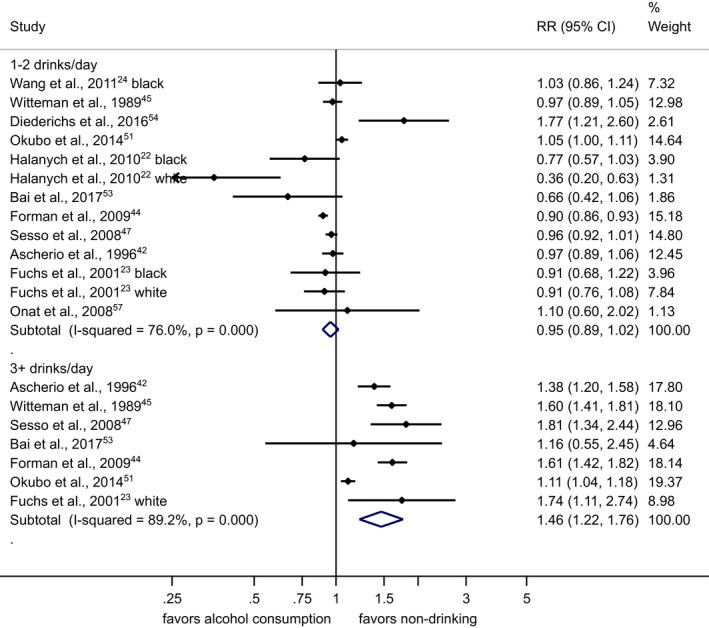
Incidence of hypertension in women by alcohol intake at baseline in standard drinks compared with abstainers in cohort studies, 1989–2017. 1 standard drink=12 g of pure ethanol per day. CI indicates confidence interval; RR, relative risk.

**Figure 4:**
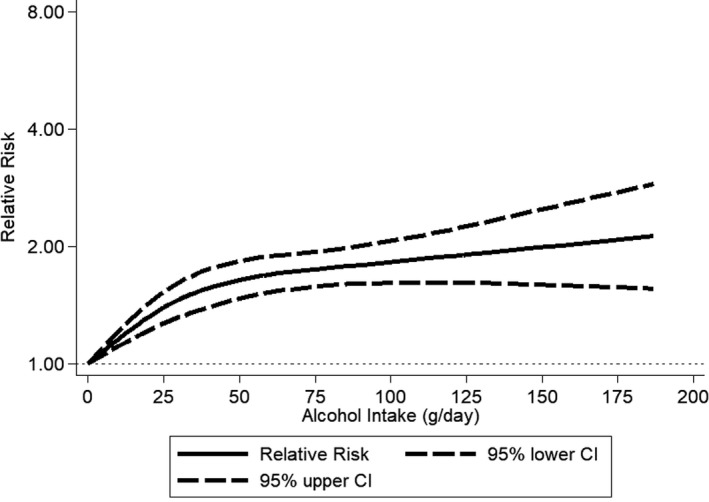
Incidence of hypertension in men by alcohol intake at baseline compared with abstainers using restricted cubic spline metaregression, 1989–2017, n=8 studies with at least 4 alcohol intake groups relative risk on the log scale. CI indicates confidence interval.

**Figure 5:**
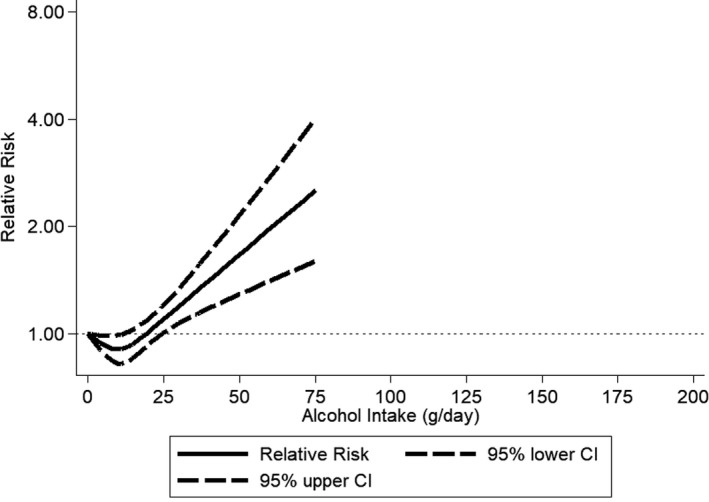
Incidence of hypertension in women by alcohol intake at baseline compared with abstainers using restricted cubic spline metaregression, 1989–2017, n=10 studies with at least 4 alcohol intake groups relative risk on the log scale. CI indicates confidence interval.

The pooled RR among former drinkers^22^, ^24^, ^50^, ^53^, ^58^ in comparison with lifetime abstainers was 1.03 (95% CI, 0.89–1.20), with virtually no differences between men and women (Figure S1). Any alcohol consumption increased the risk for hypertension compared with abstainers in men (Figure 2 and Figure S2). In women, there was no observed risk increase for consumption of 1 or 2 drinks/day in comparison with abstainers, and an increased risk beyond this level with a pooled RR=1.42 (95% CI, 1.22–1.66; I^2^=88%) for consumption of 3 or more drinks per day (Figure 3 and Figure S3). Because we included 2 studies from the Nurses’ Health Study^42^, ^45^ with different follow‐up periods of the same participants, we ran a sensitivity analysis including only 1.^42^ The results compared to Figure 3 were almost identical (1–2 drinks/day: pooled RR=0.95; 95% CI, 0.88–1.03). Different adjustment for potential confounders in regression models in primary studies resulted in little changes in RRs for different levels of alcohol consumption. In men, data for alcohol consumption beyond 75 g/d were only available from Asian countries (Figure 4). There were no data for women consuming more than 75 g/d (Figure 5). Two studies^53^, ^55^ were judged to be of serious risk of bias, 1 of low risk, and 16 of moderate risk of bias mainly because of the observational study design and 1‐time measurement of alcohol consumption (Table S2). Thirteen studies used clinical measurements of BP to determine incidence of hypertension.^22^, ^23^, ^24^, ^48^, ^49^, ^50^, ^51^, ^52^, ^53^, ^54^, ^55^, ^56^, ^57^ Similar relationships were found when we excluded studies with potential serious risk of bias and that relied on self‐reported incidence of hypertension (Figures S4 and S5).

Heterogeneity was high in most analyses, and we conducted metaregressions to investigate potential sources of heterogeneity in drinkers of 1 to 2 drinks/day. The difference between men and women was statistically significant (RR_women vs men_=0.79; 95% CI, 0.67–0.95; *P*=0.012; proportion of heterogeneity explained: 69%). There was no significant difference between studies from Asian and non‐Asian countries in men (RR_non‐Asian vs Asian_=0.93; 95% CI, 0.71–1.21; *P*=0.55) or women (RR_non‐Asian vs Asian_=0.92; 95% CI, 0.76–1.11; *P*=0.33). Three studies from the United States presented results stratified by race (black versus white). In men, people of white origin had nominally higher risk for incidence of hypertension compared with people of black origin (RR_Black vs White_=0.63; 95% CI, 0.08–4.93; *P*=0.44), whereas in women the opposite was observed (RR_Black vs White_=1.31; 95% CI, 0.66–2.63; *P*=0.38). However, the number of incident hypertension cases was low and CIs were wide, indicating low statistical power to detect significant differences. Three studies presented results by age groups.^46^, ^49^, ^51^ There was no difference in incidence of hypertension by age group (per linear increase in 4 age categories; men: RR=0.95; 95% CI, 0.84–1.09; *P*=0.43; women: RR=0.99; 95% CI, 0.86–1.13; *P*=0.83); however, statistical power was also low.

We found no evidence for small‐study bias in men or women consuming 1 to 2 drinks/day in visual inspection of funnel plots (Figures S6 and S7) or using Egger's test (*P*=0.50 and 0.38, respectively). Leaving each trial out of the analysis 1 at a time revealed no meaningful differences in effects (Figures S8 and S9).

Given the observational nature of the studies included, we rated the evidence for a causal effect of high alcohol consumption (3 or more drinks/day) on incidence of hypertension as moderate. However, evidence from randomized controlled trials^59^ support a causal effect with higher confidence. Regarding alcohol consumption of 1 to 2 drinks/day, our findings indicate effect modification by sex and no protective association. Evidence from randomized controlled trials for this level of alcohol consumption is limited and thus we judge the quality of the evidence as moderate.

## Discussion

In high‐quality cohort studies, we found that the association between average alcohol consumption of 1 to 2 drinks/day and risk of hypertension was modified by sex, with men showing an increased risk, whereas women showed no different risk compared with abstainers. Alcohol intake beyond 2 drinks/day was consistently associated with increased incidence of hypertension in both men and women.

Before discussing these results and their implications, we would like to point out some limitations. Conclusions of every meta‐analysis are determined by the quality of the original studies. This meta‐analysis is based on cohort studies, and thus this study type does not allow conclusions about causality.^60^ However, as indicated above, analogue dose‐response relationships for alcohol reduction on reduction of BP and hypertension based on trial data point to a causal effect of level of alcohol consumption on risk of hypertension,^59^ and this reasoning is corroborated by plausible biological pathways.^10^ Second, all alcohol assessments were based on subjective measurements, which may entail bias.^61^ However, most reviews come to the conclusion that subjective measurement of alcohol is reliable,^62^ even though there is some bias of underestimating true consumption, for example, sales data show higher consumption compared to survey data.^61^, ^63^ Thus, although the overall dose‐response relationship would not be affected, there may be some misestimation of the RRs of the levels of drinking (eg, previous work^64^). Finally, although patterns of drinking, in particular irregular heavy drinking occasions, have been shown to impact on BP and risk of hypertension,^65^ we could not find enough cohort studies meeting our inclusion criteria to quantify this effect by meta‐analysis.

Despite these limitations, the results are consistent in showing a dose‐response relationship between level of consumption and risk of hypertension based on observational data, corroborated by randomized controlled trials. For men, there seems to be no lower threshold, whereas for women, the dose‐response seems to emerge only beyond 2 drinks a day. For both sexes, no protective effect could be found (and was not expected given the biological pathways^10^). What could explain the differences between men and women? One explanation could be the difference in heavy drinking occasions within an overall average intake of alcohol of less than 2 drinks. An average of 2 drinks/day could be achieved by actually drinking 2 drinks every day, or by drinking 7 drinks each on Saturday and Sunday. The latter has different effects on blood pressure^66^ and thus on risk of hypertension. Further research (both observational and experimental) is necessary, however, to ascertain the effects of pattern of drinking (including peak blood alcohol level) on hypertension and thus the repeated plea to include more measures on patterns of drinking into all epidemiological work.^67^, ^68^

### Implications

Clinicians are faced with a dilemma. On the one side, low‐level drinking has been associated with less risk for ischemic heart disease^69^, ^70^; on the other side, the risk for hypertension seems increased, at least in men. In order to side with caution, patients should be advised to drink as little as possible for many reasons, including increased risk of cancer and injury, to name a few,^15^ and the risk of hypertension should be added to the list of diseases where no alcohol consumption is safe. This may require a change in drinking advice in current guidelines for prevention and treatment of hypertension, which state that men should limit their alcohol intake to 2 drinks or less, a level which we found to be associated with increased risk for hypertension. Additional research with stronger, more‐experimental study design may help in answering outstanding questions on cardiovascular risk from low levels of drinking.

Other implications of this research are clear: Alcohol consumption should be assessed at the primary care level whenever there is elevated BP.^71^ Unfortunately, despite some guidelines recommending such an approach, it is rarely followed in clinical practice (for the example of the 6 largest European Union countries^72^). Efforts should be made to change clinical practice, given that alcohol‐induced hypertension is both preventable and reversible,^10^, ^59^ and there are effective and cost‐effective interventions to reduce alcohol consumption level in primary care.^73^, ^74^ Changing clinical practice promises to reduce substantial mortality and burden of disease associated with both alcohol consumption and hypertension.^10^, ^75^

## Sources of Funding

Research reported in this publication was supported by the National Institute on Alcohol Abuse and Alcoholism (NIAAA) of the National Institutes of Health under Award Number R21AA023521 to Roerecke. The content is solely the responsibility of the authors and does not necessarily represent the official views of the National Institutes of Health. The sponsor of the study (NIAAA) had no role in study design, data collection, data analysis, data interpretation, or writing of the report. The authors collected the data and had full access to all of the data in the study. The authors also had final responsibility for the decision to submit the study results for publication.

## Disclosures

Roerecke and Rehm report grants from the National Institutes of Health (NIH), the National Institute on Alcohol Abuse and Alcoholism (NIAAA), during the conduct of the study. Rehm reports grants and honoraria from Lundbeck outside of this work (modest relationship). The remaining authors have no disclosures to report.

## Supporting information


**Table S1.** Search Strategy for Medline(R) (1946–Most Recent) and Embase (Embase+Embase Classic)
**Table S2.** Risk of Bias in Nonrandomized Studies—of Interventions (ROBINS‐I) Assessment Tool, Modified Version
**Figure S1.** Incidence of hypertension in former drinkers compared with lifetime abstainers at baseline by sex, 1989–2017.
**Figure S2.** Incidence of hypertension in men by alcohol intake in standard drinks at baseline compared with abstainers, all studies, 1989–2017.
**Figure S3.** Incidence of hypertension in women by alcohol intake in standard drinks at baseline compared with abstainers, all studies, 1989–2017.
**Figure S4.** Incidence of hypertension in men by alcohol intake in standard drinks at baseline compared with abstainers in cohort studies with clinical measurement of blood pressure and low or moderate risk of bias, 1989–2017.
**Figure S5.** Incidence of hypertension in women by alcohol intake in standard drinks at baseline compared with abstainers in cohort studies with clinical measurement of blood pressure and low or moderate risk of bias, 1989–2017.
**Figure S6.** Funnel plot for 1 to 2 drinks/day alcohol intake at baseline compared with abstainers in men, 1989–2017.
**Figure S7.** Funnel plot for 1 to 2 drinks/day alcohol intake at baseline compared with abstainers in women, 1989–2017.
**Figure S8.** Influence of omitting a single study for 1 to 2 drinks/day alcohol intake at baseline compared with abstainers in men, 1989–2017.
**Figure S9.** Influence of omitting a single study for 1 to 2 drinks/day alcohol intake at baseline compared with abstainers in women, 1989–2017.
