# Supplementary material for: Sex‐Specific Associations Between Alcohol Consumption and Incidence of Hypertension: A Systematic Review and Meta‐Analysis of Cohort Studies
Source: J Am Heart Assoc. 2018 Jun 27;7(13):e008202. doi: 10.1161/JAHA.117.008202 (PMC6064910; doi:10.1161/JAHA.117.008202)
Supplement: Supplementary file 1 — Table S1. Search Strategy for Medline(R) (1946–Most Recent) and Embase (Embase+Embase Classic) Table S2. Risk of Bias in Nonrandomized Studies—of Interventions (ROBINS‐I) Assessment Tool, Modified Version Figure S1. Incidence of hypertension in former drinkers compared with lifetime abstainers at baseline by sex, 1989–2017. Figure S2. Incidence of hypertension in men by alcohol intake in standard drinks at baseline compared with abstainers, all studies, 1989–2017. Figure S3. Incidence of hypertension in women by alcohol intake in standard drinks at baseline compared with abstainers, all studies, 1989–2017. Figure S4. Incidence of hypertension in men by alcohol intake in standard drinks at baseline compared with abstainers in cohort studies with clinical measurement of blood pressure and low or moderate risk of bias, 1989–2017. Figure S5. Incidence of hypertension in women by alcohol intake in standard drinks at baseline compared with abstainers in cohort studies with clinical measurement of blood pressure and low or moderate risk of bias, 1989–2017. Figure S6. Funnel plot for 1 to 2 drinks/day alcohol intake at baseline compared with abstainers in men, 1989–2017. Figure S7. Funnel plot for 1 to 2 drinks/day alcohol intake at baseline compared with abstainers in women, 1989–2017. Figure S8. Influence of omitting a single study for 1 to 2 drinks/day alcohol intake at baseline compared with abstainers in men, 1989–2017. Figure S9. Influence of omitting a single study for 1 to 2 drinks/day alcohol intake at baseline compared with abstainers in women, 1989–2017. [file JAH3-7-e008202-s001.pdf]

SUPPLEMENTAL MATERIAL

Table S1. Search strategy for Medline(R) (1946-most recent) and Embase (Embase+Embase Classic)

1	Human/
2	(bibliography or case reports or clinical conference or conference abstract or conference paper or conference proceeding or "conference review" or clinical trial, all or comment or congresses or editorial or guideline or in vitro or letter or meta analysis or "review" or systematic reviews).pt.
3	1 NOT 2
	Method terms
4	exp Case-Control Studies/ or case control.mp.
5	exp cohort studies/ or exp follow-up studies/ or exp longitudinal studies/ or exp prospective studies/ or exp retrospective studies/ or cohort study.mp.
6	4 or 5
	Alcohol terms
7	exp Alcohol Drinking/
8	exp Alcoholic Intoxication/
9	exp binge drinking/
10	(alcohol* adj3 (drink* or consum* or intake)).mp.
11	heavy drinking.mp.
12	alcoholic beverages/
13	OR/7-12
	Disease terms
14	hypertension/
15	high blood pressure.mp.
16	elevated blood pressure.mp.
17	hypertens$.tw.
18	exp resistant hypertension/
19	resistant hypertension.mp.
20	OR/14-19
21	3 AND 6 AND 13 AND 20
22	remove duplicates from 21

Table S2. The Risk Of Bias in Non-randomized Studies – of Interventions (ROBINS-I) assessment tool, modified version

Study	Bias due to confounding	Bias in selection of participants into the study	Bias in classification of exposures	Bias due to missing data	Bias in measurement of outcomes	Bias in selection of the reported result	Overall risk of bias
Ascherio et al., 1996[1]	++	+	+	+	++	+	Moderate
Bae et al., 2014[2*]	+++	++	+	+	++	+	Serious
Bai et al., 2017[3]	++	+	+	+++	+	+	Serious
Banda et al., 2010[4]	++	++	+	+	++	+	Moderate
Diederichs et al., 2016[5]	++	+	+	+	+	+	Moderate
Forman et al., 2009[6]	+	+	+	++	++	+	Moderate
Fuchs et al., 2001[7]	++	+	+	+	+	+	Moderate
Halanych et al., 2010[8]	++	+	+	+	+	+	Moderate
Nakanishi et al., 2001[9]	++	++	+	+	+	+	Moderate
Nakanishi et al., 2002[10]	++	++	+	+	+	+	Moderate
Niskanen et al., 2004[11]	+	+	+	+	+	+	Low
Ohmori et al., 2002[12]	++	+	+	+	+	+	Moderate
Okubo et al., 2014[13]	++	+	+	++	+	+	Moderate
Onat et al., 2008[14]	++	+	+	++	+	+	Moderate
Peng et al., 2013[15]	++	++	+	+	++	+	Moderate
Sesso et al., 2008[16]	++	+	+	++	++	+	Moderate
Thawornchaisit et al., 2013[17]±	++	++	++	++	++	+	Moderate
Wang et al., 2011[18*]	++	+	+	+	+	+	Moderate
Witteman et al., 1989[19], 1990[20]	++	+	+	+	++	+	Moderate

*Nested-case-control studies. + = low risk of bias; ++ = moderate risk of bias; +++ = serious risk of bias.
± Only the relative risk for former drinkers was used.

Figure S1. Incidence of hypertension in former drinkers compared to lifetime abstainers at baseline by sex, 1989-2017

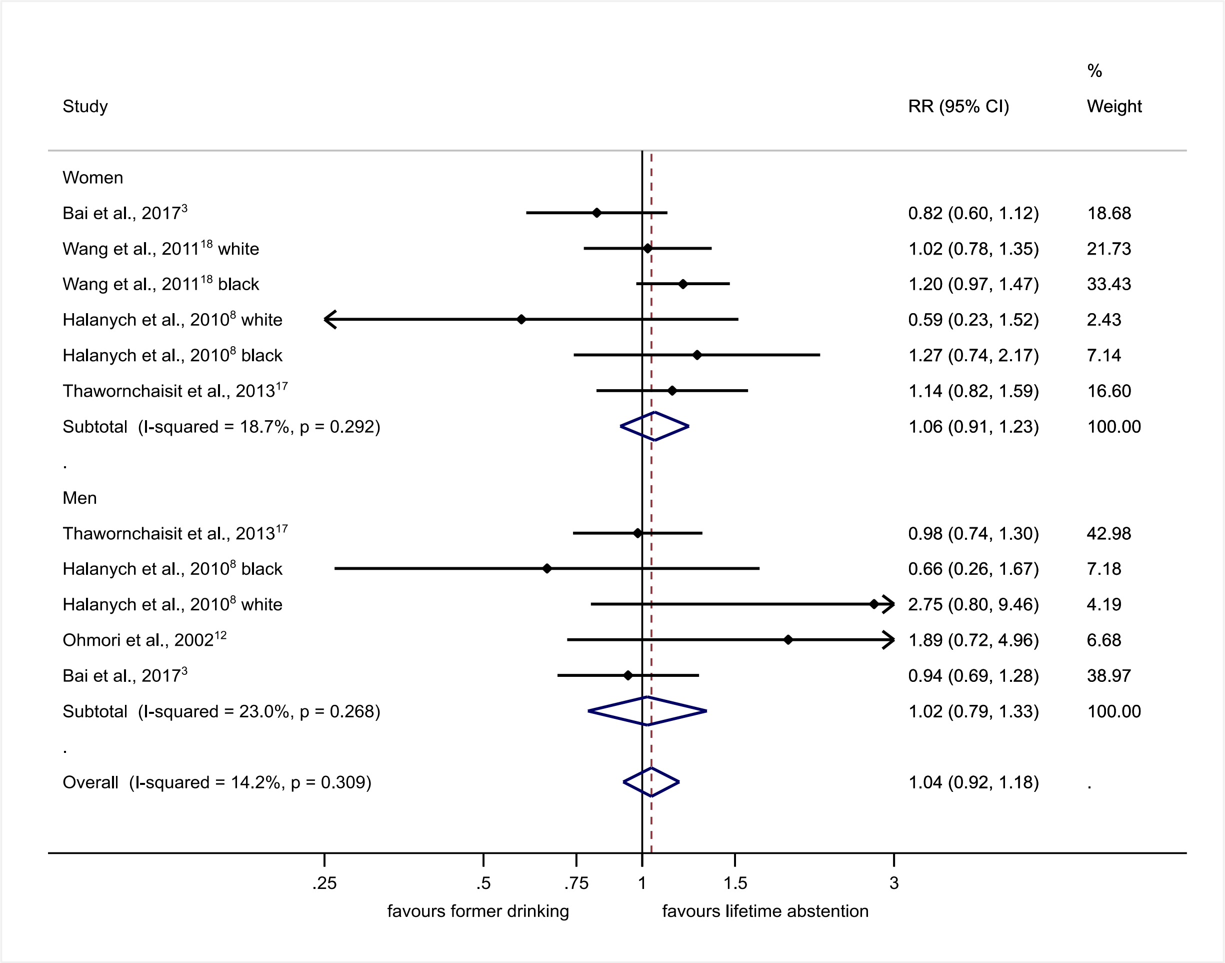

Figure S2. Incidence of hypertension in men by alcohol intake in standard drinks at baseline compared to abstainers, all studies, 1989-2017

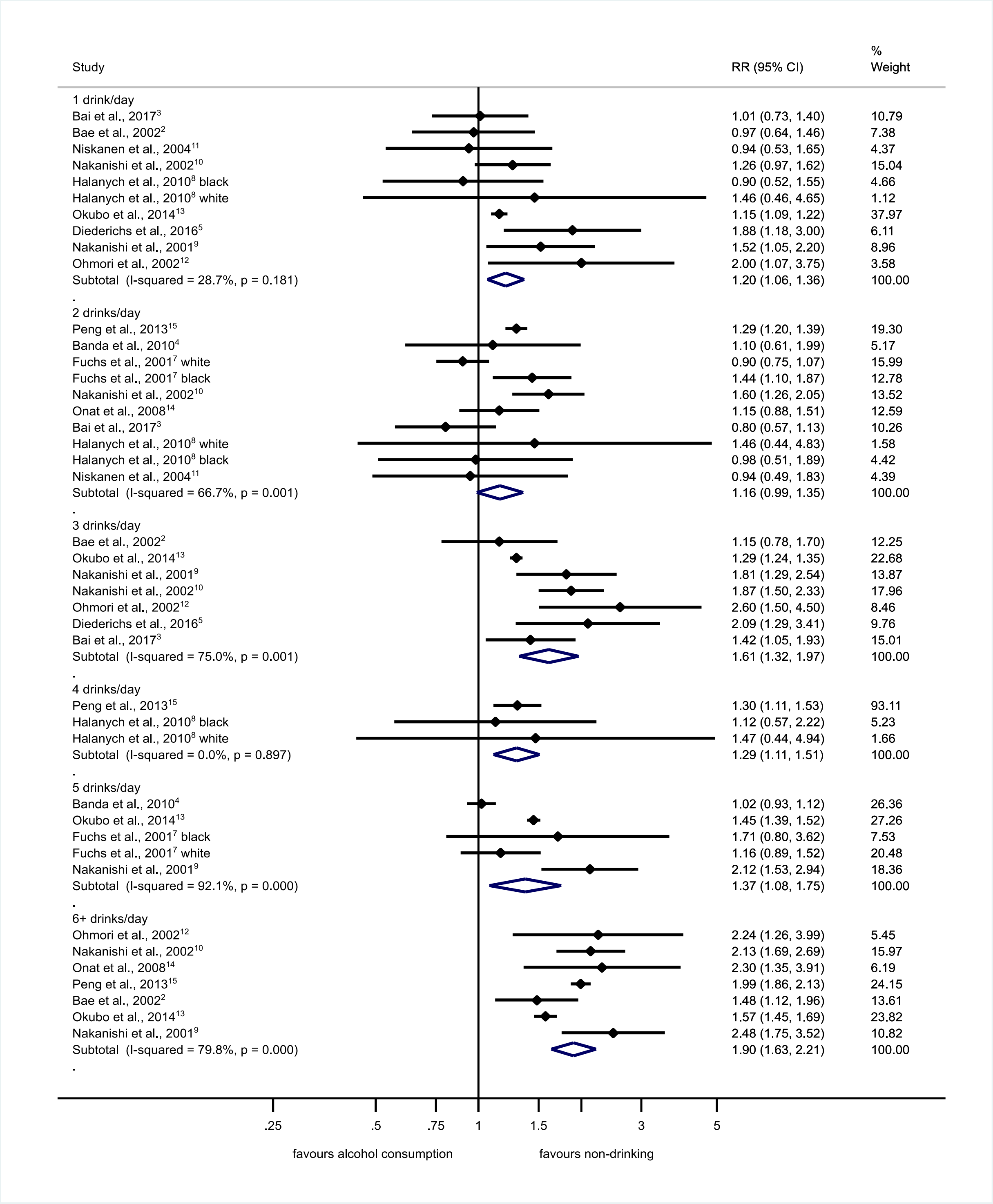

1 standard drink = 12 grams pure ethanol per day.

Figure S3. Incidence of hypertension in women by alcohol intake in standard drinks at baseline compared to abstainers, all studies, 1989-2017

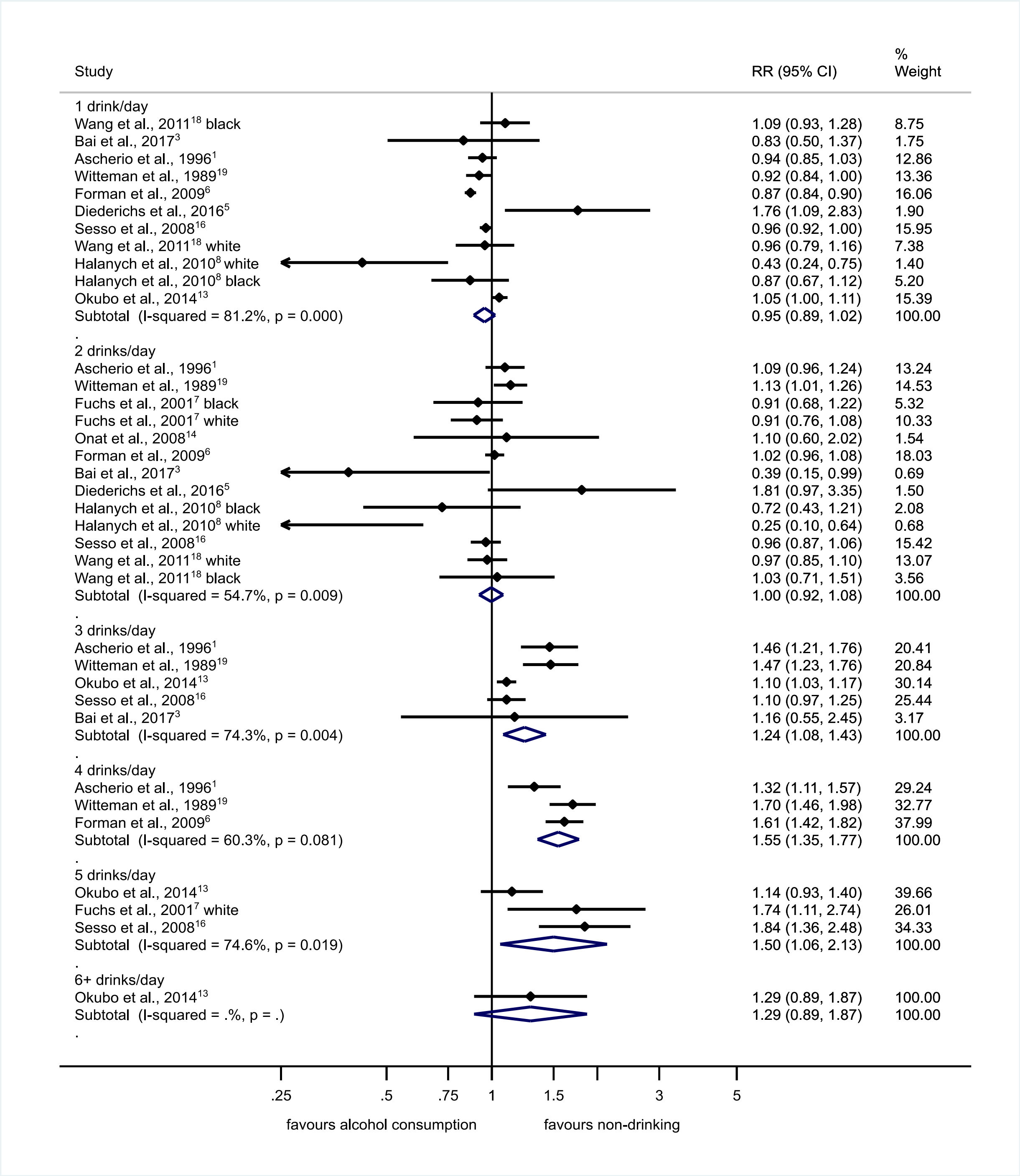

1 standard drink = 12 grams pure ethanol per day.

Figure S4. Incidence of hypertension in men by alcohol intake in standard drinks at baseline compared to abstainers in cohort studies with clinical measurement of blood pressure and low or moderate risk of bias, 1989-2017

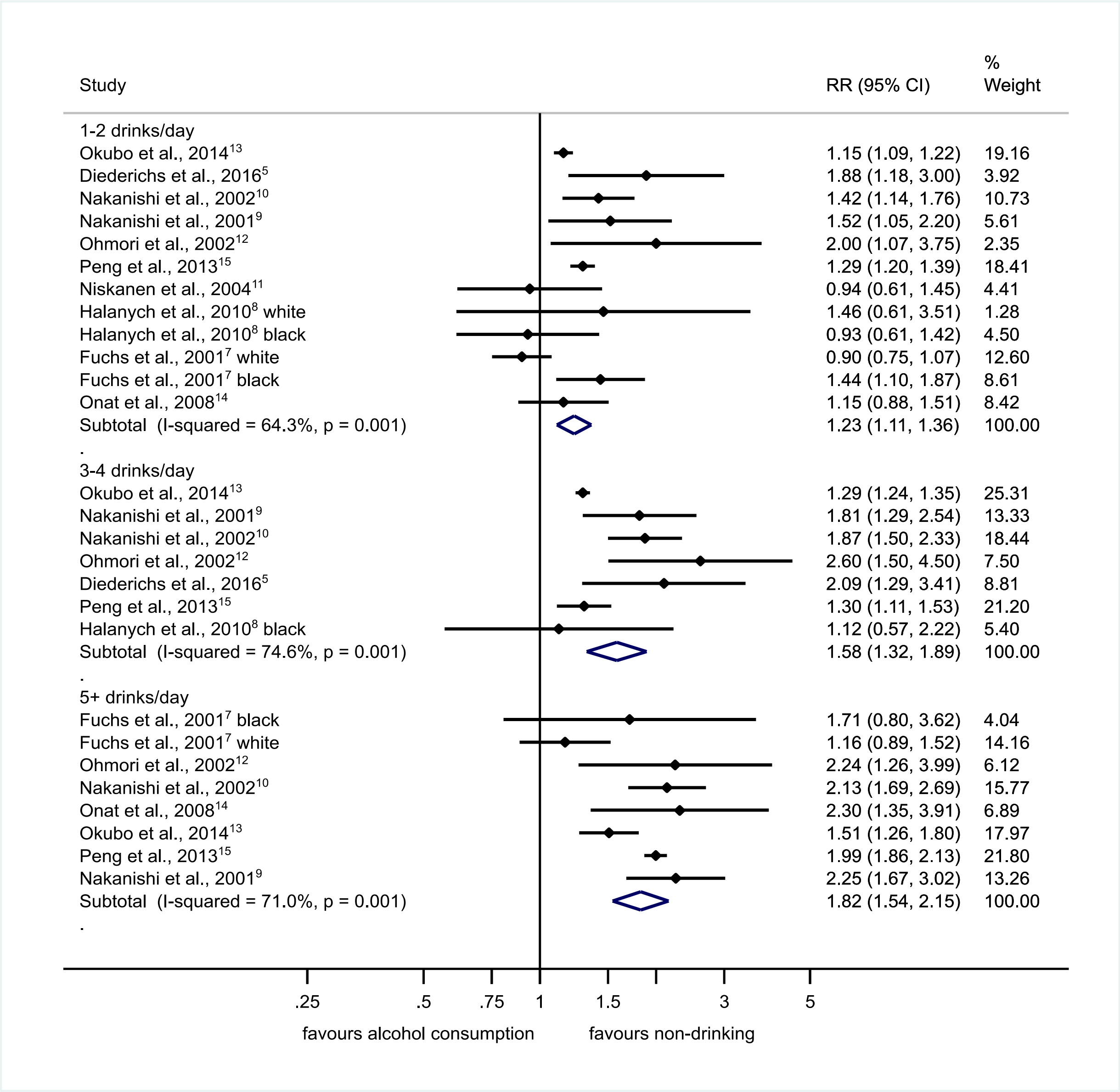

1 standard drink = 12 grams pure ethanol per day. RR = relative risk.

Figure S5. Incidence of hypertension in women by alcohol intake in standard drinks at baseline compared to abstainers in cohort studies with clinical measurement of blood pressure and low or moderate risk of bias, 1989-2017

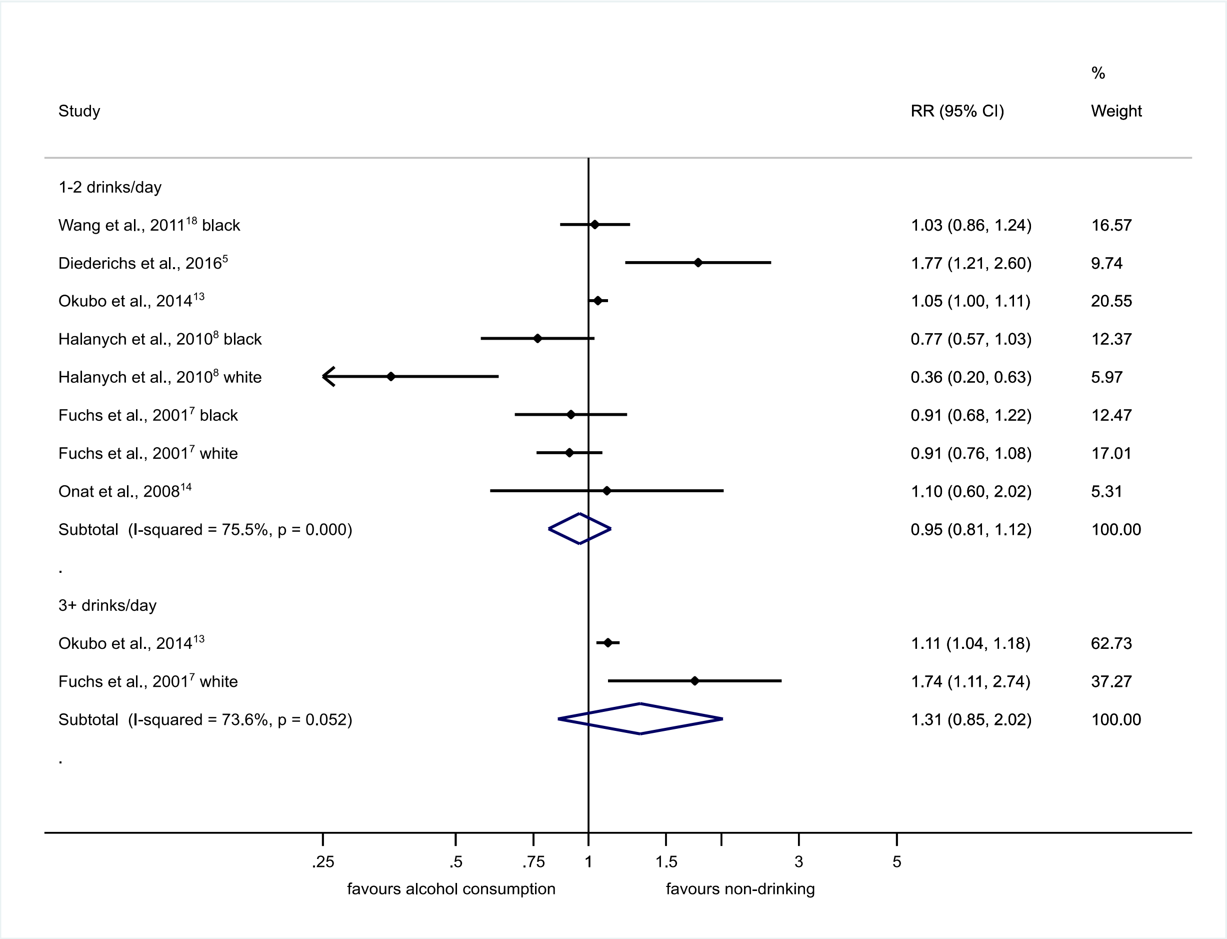

1 standard drink = 12 grams pure ethanol per day.

Figure S6. Funnel plot for 1-2 drinks/day alcohol intake at baseline compared to abstainers in men, 1989-2017

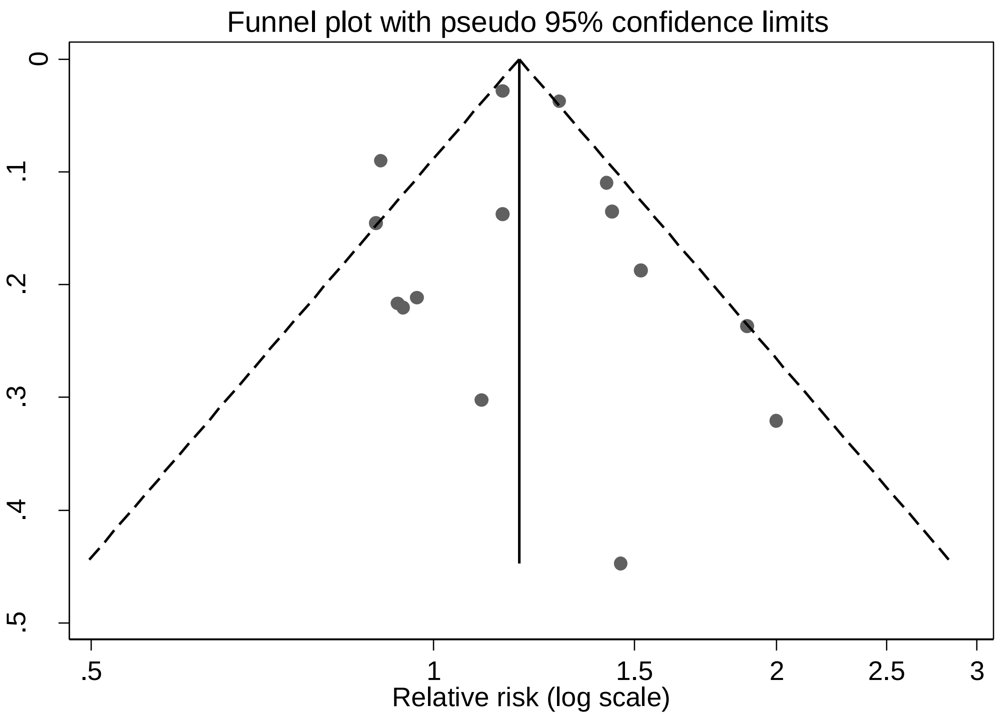

Horizontal axis shows study effects (logRR), vertical axis shows study precision (standard error of RR). Each dot represents an individual study. Vertical line shows pooled effect (random-effect model).

Figure S7. Funnel plot for 1-2 drinks/day alcohol intake at baseline compared to abstainers in women, 1989-2017

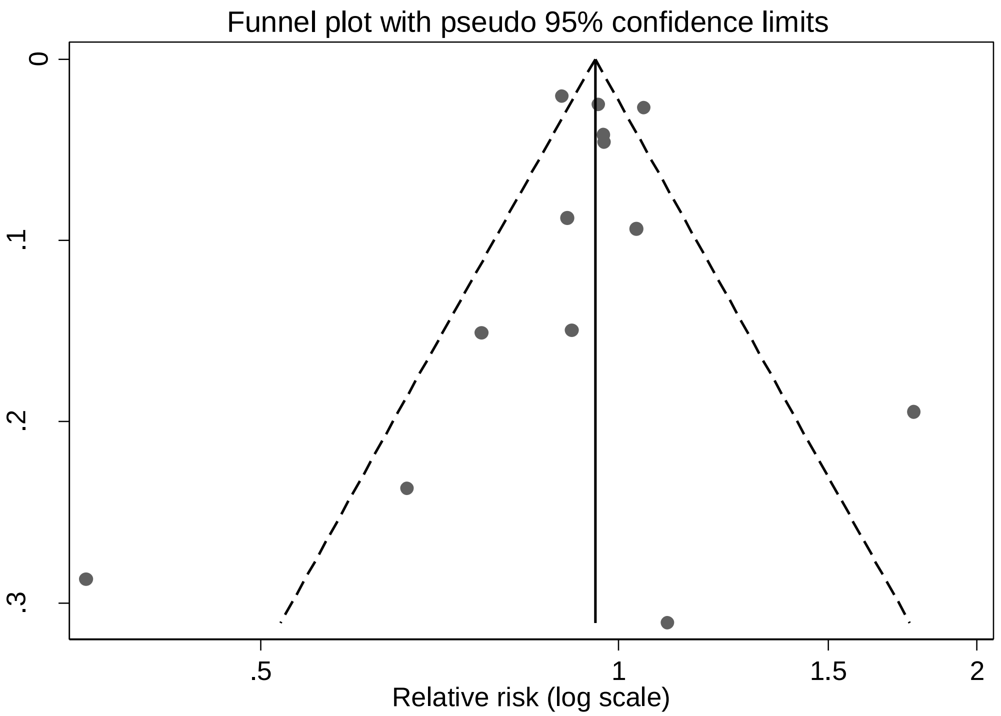

Horizontal axis shows study effects (logRR), vertical axis shows study precision (standard error of RR). Each dot represents an individual study. Vertical line shows pooled effect (random-effect model).

Figure S8. Influence of omitting a single study for 1-2 drinks/day alcohol intake at baseline compared to abstainers in men, 1989-2017

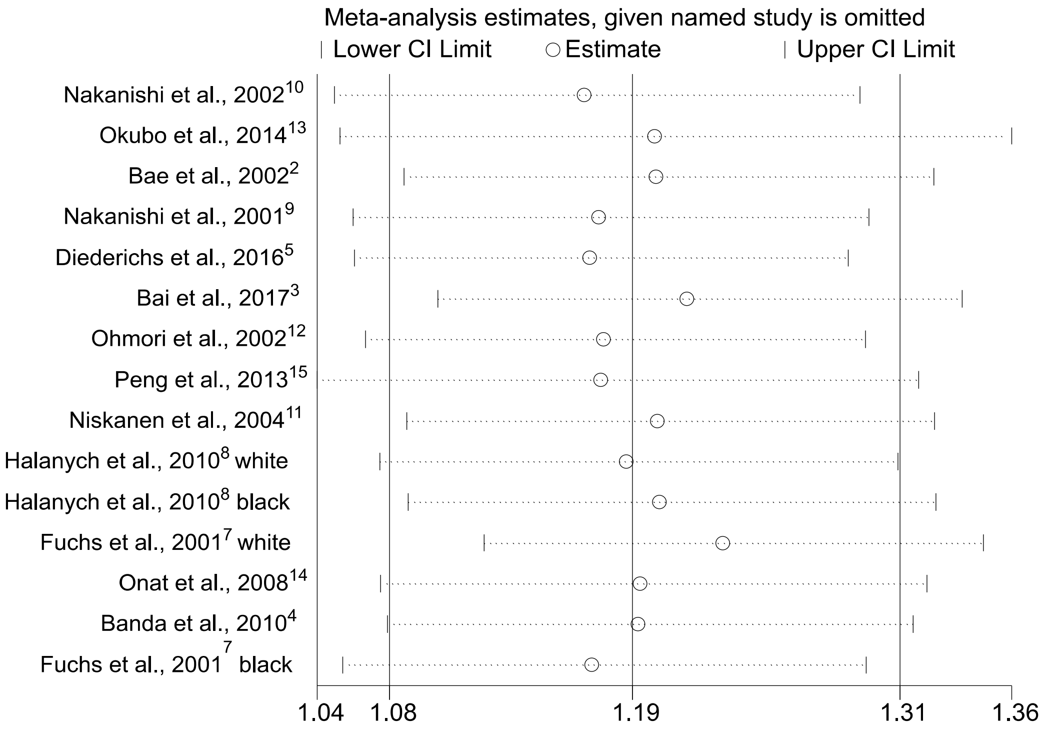

Figure S9. Influence of omitting a single study for 1-2 drinks/day alcohol intake at baseline compared to abstainers in women, 1989-2017

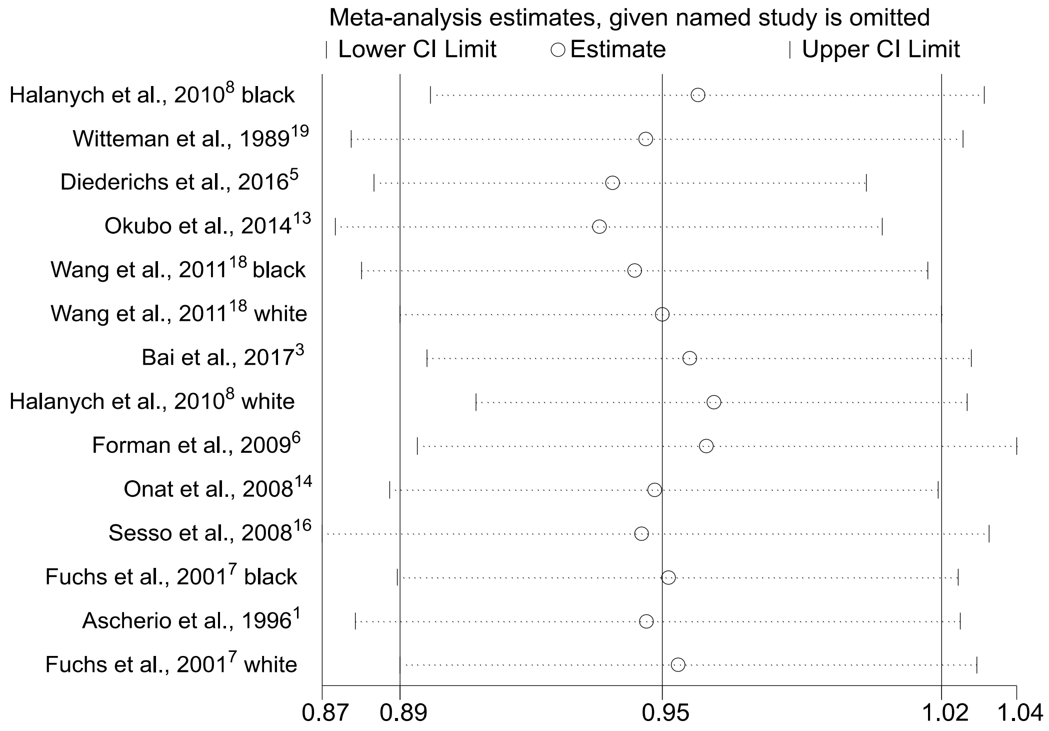

Supplemental References:

1. Ascherio A, Rimm EB, Giovannucci EL, Colditz GA, Rosner B, Willett WC, Sacks F, Stampfer MJ. A prospective study of nutritional factors and hypertension among US men. Circulation. 1992;86:1475-1484
2. Bae JM, Ahn YO. A nested case-control study on the high-normal blood pressure as a risk factor of hypertension in Korean middle-aged men. J Korean Med Sci. 2002;17:328-336
3. Bai G, Zhang J, Zhao C, Wang Y, Qi Y, Zhang B. Adherence to a healthy lifestyle and a DASH-style diet and risk of hypertension in Chinese individuals. Hypertens Res. 2017;40:196-202
4. Banda JA, Clouston K, Sui X, Hooker SP, Lee CD, Blair SN. Protective health factors and incident hypertension in men. Am J Hypertens. 2010;23:599-605
5. Diederichs C, Neuhauser H. The incidence of hypertension and its risk factors in the German adult population: results from the German National Health Interview and Examination Survey 1998 and the German Health Interview and Examination Survey for Adults 2008-2011. J Hypertens. 2017;35:250-258
6. Forman JP, Stampfer MJ, Curhan GC. Diet and lifestyle risk factors associated with incident hypertension in women. JAMA. 2009;302:401-411
7. Fuchs FD, Chambless LE, Whelton PK, Nieto FJ, Heiss G. Alcohol consumption and the incidence of hypertension: The Atherosclerosis Risk in Communities Study. Hypertension. 2001;37:1242-1250
8. Halanych JH, Safford MM, Kertesz SG, Pletcher MJ, Kim YI, Person SD, Lewis CE, Kiefe CI. Alcohol consumption in young adults and incident hypertension: 20-year follow-up from the Coronary Artery Risk Development in Young Adults Study. Am J Epidemiol. 2010;171:532-539
9. Nakanishi N, Yoshida H, Nakamura K, Suzuki K, Tatara K. Alcohol consumption and risk for hypertension in middle-aged Japanese men. J Hypertens. 2001;19:851-855
10. Nakanishi N, Makino K, Nishina K, Suzuki K, Tatara K. Relationship of light to moderate alcohol consumption and risk of hypertension in Japanese male office workers. Alcohol Clin Exp Res. 2002;26:988-994
11. Niskanen L, Laaksonen DE, Nyyssonen K, Punnonen K, Valkonen VP, Fuentes R, Tuomainen TP, Salonen R, Salonen JT. Inflammation, abdominal obesity, and smoking as predictors of hypertension. Hypertension. 2004;44:859-865
12. Ohmori S, Kiyohara Y, Kato I, Kubo M, Tanizaki Y, Iwamoto H, Nakayama K, Abe I, Fujishima M. Alcohol intake and future incidence of hypertension in a general Japanese population: the Hisayama study. Alcohol Clin Exp Res. 2002;26:1010-1016
13. Okubo Y, Sairenchi T, Irie F, Yamagishi K, Iso H, Watanabe H, Muto T, Tanaka K, Ota H. Association of alcohol consumption with incident hypertension among middle-aged and older Japanese population: the Ibarakai Prefectural Health Study (IPHS). Hypertension. 2014;63:41-47
14. Onat A, Hergenc G, Dursunoglu D, Ordu S, Can G, Bulur S, Yuksel H. Associations of alcohol consumption with blood pressure, lipoproteins, and subclinical inflammation among Turks. Alcohol.. 2008;42:593-601
15. Peng M, Wu S, Jiang X, Jin C, Zhang W. Long-term alcohol consumption is an independent risk factor of hypertension development in northern China: evidence from Kailuan study. J Hypertens. 2013;31:2342-2347
16. Sesso HD, Cook NR, Buring JE, Manson JE, Gaziano JM. Alcohol consumption and the risk of hypertension in women and men. Hypertension. 2008;51:1080-1087
17. Thawornchaisit P, de Looze F, Reid CM, Seubsman SA, Sleigh AC. Health risk factors and the incidence of hypertension: 4-year prospective findings from a national cohort of 60 569 Thai Open University students. BMJ open. 2013; 3(6) pii: e002826 doi: 10.1136/bmjopen-2013-002826

18. Wang L, Manson JE, Gaziano JM, Liu S, Cochrane B, Cook NR, Ridker PM, Rifai N, Sesso HD. Circulating inflammatory and endothelial markers and risk of hypertension in white and black postmenopausal women. Clinical Chemistry. 2011;57:729-736

19. Witteman JC, Willett WC, Stampfer MJ, Colditz GA, Sacks FM, Speizer FE, Rosner B, Hennekens CH. A prospective study of nutritional factors and hypertension among US women. Circulation. 1989;80:1320-1327

20. Witteman JC, Willett WC, Stampfer MJ, Colditz GA, Kok FJ, Sacks FM, Speizer FE, Rosner B, Hennekens CH. Relation of moderate alcohol consumption and risk of systemic hypertension in women. Am J Cardiol. 1990;65:633-637
